# Was lockdown life worth living?

**DOI:** 10.1007/s40592-022-00155-7

**Published:** 2022-03-20

**Authors:** Holly Lawford-Smith

**Affiliations:** University of Melbourne, Melbourne, Australia

**Keywords:** COVID-19, Lockdowns, Domestic violence, Financial stress, Life worth living, Parfit

## Abstract

Lockdowns in Australia have been strict and lengthy. Policy-makers appear to have given the preservation of quantity of lives strong priority over the preservation of quality of lives. But thought-experiments in population ethics suggest that this is not always the right priority. In this paper, I'll discuss both negative impacts on quantity of lives caused by the lockdowns themselves, including an increase in domestic violence, and negative impacts on quality of lives caused by lockdowns, in order to raise the question of whether we each had reason to choose quantity over quality in our own lives in a way that would justify the lockdowns we had.

In population ethics, the focus has generally been imagined future populations. Philosophers discuss the value of continuing the lives already in progress rather than replacing them with new lives; whether existence itself is a harm, neutral, or a benefit; what a life has to be like for a person to be indifferent between life and death; the things that make life worth living; and how to make trade-offs between quantity (the number of people in a population) and quality (the quality of life each person in a population has). The motivation has been, variously, challenges to the consequentialists’ ideal of the greatest wellbeing for the greatest number, and reflections on what we owe to future generations, especially in light of climate change.

But population ethics is not only about imagining future populations in the service of refining our moral principles for the current population’s use. Policy-makers have been doing a kind of population ethics for the past two years, since the COVID-19 pandemic begin. Their trade-offs have not been between quality of life and increases in population size through birth or immigration, but rather between quality of life and the prevention of decreases to the population by way of COVID-19 deaths. They have had to make decisions about the badness of existing lives not continuing, or of being shortened from what they otherwise would have been, and the badness of subjecting people—people of various levels of vulnerability—to the risk of death or serious disease (e.g. long-COVID), compared to the reduction of people’s quality of life caused by lockdowns. Both are trade-offs between quantity and quality of life in a population.

Because population ethicists are usually thinking about increases (population growth), stability (population replacement), or decreases (population reduction) being a matter of people having greater or fewer numbers of children, or the state accepting greater or fewer numbers of immigrants, there is more space for ambivalence about the respective values of quantity and quality. We can ask whether resources are so scarce that a hypothetical baby would create additional pressure that reduces everyone else’s share, without feeling that we are wronging the baby. But these questions look more callous when we are talking about existing people, wondering whether it’s permissible to implement policies that will predictably end or shorten some of their lives, in order that other people’s wellbeing not be reduced.

Perhaps this explains why, at least in Australia, lockdowns have been so strict and so lengthy. We are not, really, trading off between quantity of lives and quality of life. Rather, we are giving the preservation of existing quantity—the continuation of existing lives—strong priority over quality. Significant reductions in quality of life are justified on the basis that they protect against a reduction in quantity of lives.^1^

There are two complications, however. One is that policy-makers have tended to focus on the risk of death and disease caused by COVID-19, but not the risk of death and disease caused by the proposed mitigation strategy, namely lockdowns^2^ (as Peter Godfrey-Smith argues persuasively, in this issue).^3^ That means the trade-off is not quality versus quantity, in all cases, but sometimes *quantity* versus quantity, with one set of impacts on quantity being largely overlooked. I will elaborate on these in Sect. 2. The other is that it’s not clear that the strong priority given to quantity over quality is really justified. At least in the abstract, philosophers have shown that there are situations in which quality should be preferred (the world imagined in Derek Parfit’s Repugnant Conclusion, which I’ll talk more about below, is one example). Thus I want to pick up on and extend the project Godfrey-Smith started in his paper, of looking more closely at what reductions in quality of life have been imposed on which groups of people, and whether they can be said to have gotten us closer to the point at which quality should start to have priority over quantity than policy-makers have acknowledged. I’ll do this in Sect. 3. In Sect. 1, I’ll say a bit more about some of the population ethicists’ useful discussions.

Lockdowns are temporary, and as I write this paper in November 2021, they seem to be ending. If lockdown life were just *life* (with no hope of parole) the considerations adduced in Sects. 2 and 3 would likely provide an argument for resisting the imposition of lockdowns. But that they have been, and may yet turn out to still be, *part of life*, gives the following discussion a slightly different role. It asks us to start taking seriously the question of when the costs of lockdowns would start to outweigh their benefits; whether we’ve been working with a full enough understanding of what the costs and benefits actually are; and whether we’ve been assigning the right weighting to those respective costs and benefits. (Specifically, whether we have been overweighting deaths from COVID-19 and underweighting death and disease from other causes, and/or underweighting particular negative impacts on quality of life).

## Quality vs. quantity between populations

Parfit (1984) introduced a set of cases designed to test our intuitions about the number of people in a population versus those people’s quality of life. In one, he compares two populations, *A* and *B*. *A* is a small population with very high quality of life. *B* is twice the size of *A,* and has about two-thirds of *A’s* level of wellbeing. In each population, quality of life is equal between individuals (“no one is worse off than anyone else”) (Parfit 1984, p. 385).^4^ While the individuals in *B* have lower quality of life than the individuals in *A,* their quality of life is still high: “it would take much more than another similarly great decline before people’s lives ceased to be worth living” (*ibid*). In giving some texture to *B’s* comparatively lower wellbeing, Parfit suggests “There might be worse housing, overcrowded schools, more pollution, less natural beauty, and a somewhat lower average income” (*ibid*).^5^*B*’s greater size also leads to *B’s* having a greater total population wellbeing than *A.* So if we care about total population wellbeing, we might think that *B* is better than *A.* The quality of life is lower, but it is still good, and more people get to have the lives that are worth living.

Parfit shows that we can repeat this exercise (introducing population *C,* double the size of *B* and with lower quality of life but still lives worth living; and so on) all the way to a population that is huge and all of whose members have lives just barely worth living. But many people’s intuitions flip at this point, or somewhere before it: a smaller population of people with higher quality lives is *better* than a larger population of people with lower quality lives. This is a strike against the utilitarian, because it is not total population wellbeing that determines which population is more desirable from a moral point of view. Parfit calls the end of the sequence—the largest population with lives “not much above the level where life ceases to be worth living” (*ibid,* p. 388)—the Repugnant Conclusion. He thus shares the intuition that quantity does not have priority over quality.

However, Parfit’s populations were only imagined, and in the sequence from *A* through *B* and *C* all the way to the Repugnant Conclusion, he simply stipulated a fixed population size double the one before it. When we move to apply the intuition many people have about the Repugnant Conclusion to our own population, quantity operates differently. We might bring a new person into existence (population increase by birth), or add someone from another population into our own (population increase by immigration). An existing person might die (population decrease by death), or might move into another population than our own (population decrease by emigration). Even if we all agree that a population with fewer people who have higher quality of life is preferable to a population with greater numbers of people who have much lower quality of life, that wouldn’t justify deliberately *moving* from the latter to the former at a time, or within a short period of time. The available means for doing so are morally prohibited, e.g. forced deportation,^6^ killing, and letting die.

Facing COVID-19, with Australia’s international border closed, there is no question of change to population size via immigration/emigration. The quality/quantity issue is the narrower one of *preventing deaths from COVID-19*^7^ vs *maintaining quality of life* (or, not reducing it too substantially). That means there is also no question of increase to population in order to increase total population wellbeing through the number of people with lives worth living. All that is at issue when it comes to quantity is the preservation of existing lives. It may be that people’s intuitions about quality vs. quantity would change when what’s at issue is maintenance of an existing population rather than increase to population. Increases in Parfit’s hypothetical populations add ‘strangers’ who could have no prior social ties, and no right to exist. Decrease in our actual population subtracts existing people, embedded in webs of social relationships, and—at least arguably—with a right against preventable death.

Here we hit our first difficult conceptual question. Say that instead of lockdowns, the Australian federal and state governments simply let COVID-19 run its course, and this led to significantly more deaths. In this scenario, what obligation did the respective governments have to those who died, and in what way did they fail in those obligations? If each resident^8^ had a right that they not be let die, then the relevant government failed in a negative obligation: the proximate cause of death was the virus, but the death was predictable and preventable, and the government had an obligation to prevent it, so it can be classed as a ‘letting die’ by the government. Even though the death happens by way of an ‘omission’ or ‘allowing’ rather than an ‘acting’ or ‘doing’, responsibility lies with the government. Alternatively, if each resident had only a claim of some kind to be saved/rescued, then the relevant government failed only in a positive obligation. The virus threatened some people with death, and the government could have stepped in to rescue them, and it would have been good if it had done this; but it was not required, especially when there are so many other things competing for its attention and resources.

Negative obligations are generally taken to be a lot stronger than positive obligations, so in a contest for priority quality might do better against quantity when it is a matter of positive obligations to preserve life (by saving/rescuing) than a matter of negative obligations not to destroy life (here by letting die). Suppose the government has only positive obligations to preserve life. In Australia, the majority of deaths from COVID-19—59%—occur *after* the average age of life expectancy. Life expectancy is 82.9 years,^9^ currently 80.9 years for males and 85 years for females.^10^ Of the 2020 deaths, 24% were in the 85–89 age cohort, and 35% were in the 90 + cohort (AIHW 2021, p. 33), and 75% of those who died were living in residential aged care facilities (*ibid,* p. 25). One might argue that there is no obligation to preserve life after the age of life expectancy; or more weakly, that the strength of the obligation to preserve life after the age of life expectancy is reduced. (This does not directly affect precautionary policy aimed at preventing deaths from COVID-19, given that 41% of those deaths are in cohorts prior to life expectancy. But it may affect the cost/benefit calculations—at least if quantity is not given so much weight that it in practice appears to have lexical priority—by reducing the numbers of people likely to be impacted). Suppose, on the other hand, that the government has negative obligations not to let people die. Letting die is very bad, so this is likely to justify giving priority to quantity (preservation of the existing population) over quality. Whether we think the obligations are negative or positive may depend on the character of the particular population’s government, and the expectations of the people who have lived under it. I will not attempt to resolve this question here; only raise it for consideration.

It will be helpful to get a clearer idea about lives worth living, and what contributes to increases in the quality of life, in order to know how to think about this abstract trade-off between quantity of people and quality of lives. In a (2016) paper, Parfit says more about these things, and argues for giving lexical priority to quality, not quantity, of lives.^11^ Quality of life includes length of life, proportions of good and bad things, and the number and type of good things (Parfit 2016, p. 118). Examples of good things include “Mozart’s music”, and “Venice” (Parfit [1986] 2004, p. 19), and (by implication) high quality housing, uncrowded schools, unpolluted air, pristine natural landscapes, ample opportunities (*ibid*, pp. 7–8), and (again by implication, and perhaps more controversially) equality (*ibid,* p. 11). A very short life; a life that ‘roller-coasters’ between good and bad things, containing lots of bad even if also lots of good; and a life that is ‘drab’, containing “very little that was good” (Parfit 2016, p. 118); are all lives of low quality. To illustrate drab lives, Parfit imagines that the only good things are “muzak and potatoes” (*ibid*).^12^ Life for this population is very unlike our own: no art galleries (no art); no music concerts (no music but muzak); none of the range of foods and flavours (no food except potatoes); no dancing, no poetry, no literature, no movies, no sports, no podcasts—you get the picture.

Parfit does not say much about lives not worth living. He gives one dramatic example, that he calls ‘Hell’: “In this outcome the extra group are innocent people who all have lives which are much worse than nothing. They would all kill themselves if they could, but their torturers prevent this” (Parfit [1986] 2004, p. 12). Aside from this, the details are left unclear. Critics have pushed back by saying that virtually any kind of life, no matter how lacking in good things and full of bad things, is worth living. Partha Dasgupta, for example, notes that even the “wretched of the earth”—the “hundreds of millions of… people” who are “disenfranchised, malnourished, and prone to illness, but surviving”—“tenaciously display[…] that their lives are worth living by the persistence with which they continue to wish to live” (Dasgupta 1994, p. 116, quoted in Mulgan 2002). It’s not clear, though, that we should conclude from the fact that people don’t consider killing themselves, or don’t actually kill themselves, despite the wretchedness of their circumstances, that their lives are worth living. Suicide is difficult, and painful, and (normally) inflicts serious costs on other people. It is a cultural taboo. It remains an open question, I think, what makes a life not worth living, and how many current lives fall below this level.^13^

Parfit applies the quality over quantity intuition *within* an individual person’s life, as well as between populations. He considers two futures for himself, one in which “I could live for another 100 years, all of an extremely high quality. Call this the Century of Ecstasy” (Parfit [1986] 2004, p. 17), the other in which “I could instead live for ever, with a life that would always be barely worth living. Though there would be nothing bad in this life, the only good things would be muzak and potatoes. Call this the Drab Eternity” (*ibid,* pp. 17–18). He judges that the Century of Ecstasy is a better future than the Drab Eternity, and notes that “Many people would have the same belief, and preference” (Parfit [1986] 2004, p. 18). Drab days do not add up in a way that means enough of them would at some point begin to outweigh the value of ecstatic days. The higher quality of life has lexical priority over the larger quantity of life, because no amount of the latter can compare to some amount of the former. This within-life application will be important for us: did each of us have reason to accept a reduced risk of our lives being ended, or shortened, *in exchange for* many of the things that made those very lives worth living?

## Quantity vs. quantity within locked-down populations

In his paper ‘Covid Heterodoxy in Three Layers’ (this issue), Peter Godfrey-Smith writes:

I say that an intense focus on immediate medical harms [of COVID-19] is sidelining consideration of more scattered, diverse, and longer-term harms arising from shuttered businesses, disrupted educations, alienation, and the like. …those harms will be harder to track and quantify, and often more inherently unpredictable as they involve long causal paths that wind through networks affected by other factors. [But] … Uncertainty about longer term harms does not make them smaller or less important (Godfrey-Smith 2021 p. 5).^14^

Some of the harms he has in mind are reductions in quality of life, which I will take up in Sect. 3. Others are harms to quantity of life, impacts on mortality and morbidity, caused by lockdowns themselves.^15^ Godfrey-Smith notes that “While pessimistic scenarios on the health side are made very salient, pessimistic scenarios on the other side are rarely seen on the table” (*ibid*). One of his examples is the longer-term impacts of “suddenly expanding the educational gap between wealthy and poor children, owing to the greater ability of rich families to keep their kids’ education going through public school closures”, and worries that these impacts could be “catastrophic” (*ibid*).

One measure for tracking mortality impacts of COVID-19 is “excess deaths” relative to some baseline, but this measure will include *both* deaths from COVID-19 and deaths from “the effects of lockdowns and related policies themselves” (*ibid,* p. 3). Deaths from lockdowns (or with lockdowns as a contributing cause) will become virtually impossible to track over the longer-term, as they are folded into ordinary mortality statistics.

In this section I’ll focus on three main sets of impacts on quantity of life caused by lockdowns. (Where Godfrey-Smith’s focus was largely on the ongoing effects *of* lockdowns, my focus will be largely on the effects *within* lockdowns). The first is the increase in domestic abuse affecting mostly women. The second is the increase in severe stress caused by economic insecurity. The third is the long-term health impacts of increased consumption of alcohol, and weight gain.

### Domestic abuse^16^

In April 2020, *The New York Times* published an article titled ‘A New Covid-19 Crisis: Domestic Abuse Rises Worldwide’, reporting on a ‘worldwide surge in domestic violence’. In its opening paragraph it say ‘Mounting data suggests that domestic abuse is acting like an opportunistic infection, flourishing in the conditions created by the pandemic’ (Taub 2020). There had been surges in domestic abuse in countries including China, Spain, Italy, Britain, Brazil, Cyprus, the United States, and France: an 18% increase in the first two weeks of lockdown in Spain; a 20% increase in two counties, Avon and Somerset, a week into lockdown in Britain; and a 30% spike in France^17^ (*ibid*). In one county in Jingzhou, China, reports of domestic violence in February 2020 had tripled relative to the same month of the previous year (Wanqing 2020). In the United States, Portland saw a 22% rise in arrests relating to domestic violence, and there was a 27% increase in calls to police about domestic violence in Jefferson County, Alabama (Sharma and Borah 2020). A judge in Rio de Janeiro who specialises in domestic violence told *The Guardian* that the estimated increase was “40% or 50%”, and in Cyprus, calls to a domestic violence hotline went up 30% in the week following the island’s first case of COVID-19 (Graham-Harrison et al. 2020).

In Chicago, calls to one of the city’s domestic violence hotlines went from 383 to 549 between early March and late April 2020 (Bosman 2020). Callers had asked for advice on keeping partners calm, saving money in secret, and developing code words with their children so that the children would know to call the emergency services (*ibid*). A shelter in New York city serving mainly Latin American women had a 35% increase in calls to their help line between February and March 2020 (Southall 2020). The number of calls dropped in Los Angeles and New York, but authorities said they “believed that victims were in such close quarters with their abusers that they were unable to call the police” (Bosman 2020; see also Southall 2020; Stone et al. 2020). This belief is supported by the fact that in Italy, “calls to helplines had dropped sharply, but instead they were receiving desperate text messages and emails” (Graham-Harrison et al. 2020).

No such increases were reported in Australia (see Boxall et al. 2020, p. 2 & references therein). However, in May 2020, the Australian Institute of Criminology surveyed 15,000 Australian women about their experiences of domestic violence in the three months prior. 51.8% of the women surveyed had been in cohabiting relationships for at least some part of the twelve months prior to the survey, and for 95.7% that was the same relationship they were still in when they completed the survey (*ibid,* p. 5). The Institute found that 4.6% of the women surveyed had experienced physical or sexual violence (4.2% physical violence and 2.2% sexual violence) from a cohabiting partner (current or former) in the three months before the survey; that 5.8% had experienced coercive control^18^; and 11.6% had experienced one or more of emotional abuse, harassment, or controlling behaviour (*ibid*, p. 1 & pp. 6–7). They also found that two-thirds of the women who had experienced physical or sexual violence “said the violence had started or escalated in the three months prior to the survey” (*ibid,* p. 1). When the pool of respondents was limited to those who had been in the same relationships for the last 12 months,^19^ the numbers went up significantly: 8.2% of those women experienced physical violence; 4.2% experienced sexual violence; 11.1% experienced coercive control; 22.4% experienced emotional abuse, harassment, or controlling behaviour (*ibid,* p. 6). If we take physical violence, sexual violence, and coercive control together, then 6.8% of the women surveyed, and 13.2% of the women in the same relationships for the last twelve months, had experienced at least one of these (*ibid,* p. 7).

Domestic abuse can include physical violence, “isolation from friends, family, and employment; constant surveillance; strict, detailed rules for behaviour; and restrictions on access to such basic necessities as food, clothing and sanitary facilities” (Taub 2020). The domestic violence shelter Esperanza United, in Minnesota, United States, considers domestic violence to include ‘psychological or emotional abuse (threats, insults, and put downs)’; ‘Physical abuse (hitting, kicking, punching, choking)’; ‘Economic abuse (controlling the money, taking your paycheck, stalking or harassing you at your job, or getting you in trouble with your boss’; ‘Sexual abuse (forcing sex or sexual acts, or forcing you to watch sex acts)’; and ‘Intimidation (threatening to take away children or kick you out of the house, throwing things or punching walls, harming pets, threatening to harm children, loved ones or prized possessions abroad’).^20^

Lockdowns not only give abusers more control over their victims, but also make it harder for victims to escape or turn to their regular networks for support. It was already well-known, prior to lockdowns, that ‘Domestic violence goes up whenever families spend more time together, such as the Christmas and summer vacations’, and that ‘abusers are more likely to murder their partners and others in the wake of personal crises, including lost jobs or major financial setbacks’ (Taub 2020). These personal crises are made more frequent by the COVID-19 pandemic and the policy measures responding to it. The article criticizes governments for their failure ‘to prepare for the way the new public health measures would create opportunities for abusers to terrorize their victims’ (*ibid*).

There are impacts not only on the women who are the majority victims of abusers, but on their children. There was a 17% increase in calls and texts to a national child abuse hotline in the United States in April 2020 compared with the same month in the previous year (Bosman 2020), and a spike in the numbers of child abuse cases at a hospital in Fort Worth in March 2020 (*ibid*).

One complication is that even if numbers of individual women and children experiencing abuse did not increase, the ‘intensity and frequency of abuse’ might, “a pattern that experts witnessed during the economic downturn of 2008 and immediately after 9/11, Hurricane Sandy and Hurricane Katrina” (Gupta and Stahl 2020).

What are the health impacts of domestic abuse? There are both psychological and physiological impacts. Teresa Burns, manager of the shelter Esperanza United mentioned above, spoke to *The New York Times* about victims living under lockdown having no “window of relief” from their abusers. She said “When the mind is constantly in fight, flight, freeze [mode] because of perpetual fear, that can have a lasting impact on a person’s mental health” (Gupta and Stahl 2020, their addition).

The Australian Institute of Health and Welfare reports on the health impacts of domestic abuse. They start with a 2015 study estimating the disease burden caused by domestic abuse.^21^ They focused on “the amount of disease burden that could have been avoided if no female aged 15 and over in Australia in 2015 were exposed to intimate partner violence” (AIHW 2020).^22^ They found causal links between domestic abuse and six diseases. These were depressive disorders, anxiety disorders, alcohol use disorders, early pregnancy loss, homicide and violence (the latter understood as injuries due to violence), and suicide and self-inflicted injuries” (*ibid*). They found that absent domestic abuse, there would have been 41% less female-victim homicide and violence, 18% less early pregnancy loss, 19% less suicide and self-inflicted injuries, 19% less depressive disorders, 12% less anxiety disorders, and 4% less alcohol disorders. Overall, domestic abuse was thought to have contributed to 223 deaths, and 1.6% of the total disease and injury burden, for 2015 (*ibid*). In 2017–2018, 6500 out of 21,300 hospitalisations for assault (almost 31%) in Australia were caused by family and domestic violence, with 4,800 of those people being female (73%) and 1700 being male (27%). 292 of the females were pregnant (*ibid*).

If the incidence, frequency, and severity of domestic abuse increases during lockdowns, then there will be a corresponding increase in these negative impacts on the overall disease burden. These are ‘quantity of life’ issues, and yet they were not factored into decision-making about locking down to avoid the COVID-19 -specific mortality and morbidity risks.

### Work & financial stress^23^

The Australian Bureau of Statistics tracked the impacts of COVID-19 and policy responses to it on Australian jobs and businesses in the year following restrictions first being announced (March 2020–March 2021). In March 2020, 66% of businesses reported a reduction in turnover and 64% reported a reduction in demand. In April 2020, hospitality jobs decreased by 35%, and underemployment hit a historic high of 13.8%, with 1.8 million Australians working either reduced hours, or zero hours. By May 2020, 870,000 people had lost their jobs, and 72% of businesses saw their revenues decreased. In July 2020 unemployment was 7.5%, the highest it had been in more than 20 years. By November 2020, there had been 64,000 jobs lost in retail and hospitality management, 64,000 jobs lost in hospitality, and 43,000 jobs lost in sports and personal services (ABS 2021).^24^

Young women were more likely than men to have lost their jobs due to COVID-19: 45% of women aged 18–24 lost their jobs, compared to 34% of men in the same age bracket. More young women than men reported “high levels of mental distress”, too: 24% of women compared to 21% of men. This “likely reflect[s] women’s greater representation in the industries directly affected by COVID-19, and increased caring responsibilities during the pandemic” (Kabatek 2020). Unemployment affected the age group 18–24 the worst, having the sharpest decline between March and April 2020, and remaining at the lowest rates from March through September 2020 (*ibid*). Employment rates were worst for Victorians aged 18–24, recovering from May 2020 in the rest of Australia, but worsening from June 2020 for Victorians (*ibid*).

All of those affected by job losses or reduced hours are likely to have expenses—rent or mortgages, bills, food and other essential goods. This is a significant source of stress, worrying about how to make payments. Even with government support programmes like JobKeeper and JobSeeker, the relief payments may not have been sufficient to cover an individual’s existing financial commitments.^25^ Those in the gig economy—e.g. Uber drivers—are likely to have suffered stress related not only to reduced work but also specifically related to the uncertainty of the available work. Godfrey-Smith writes “It is not “economic libertarianism” to think that forcing someone to shut down [their business] in a situation with totally inadequate safety nets is more than an ordinary financial harm. The economic context in which they operate is one in which their freedom to continue trading is integral to getting by (not being evicted from their home, and so on)” (Godfrey-Smith 2021 p. 14).

Even those who didn’t lose their jobs or have their work hours reduced experienced work-related stress. An August 2020 report by the Melbourne Institute noted that the COVID-19 pandemic had introduced “two major sources of mental distress: financial stress, and stress caused by work-family-conflict” (Broadway et al. 2021, p. 2).^26^ Experiencing job loss or reduced hours could cause distress to the individuals affected, and could also cause stress to partners (e.g. because a secondary earner becomes the sole earner) (*ibid*). Fathers were the most impacted; prior to the pandemic only 5–9% reported mental distress at high levels, and this increased during the pandemic to 25% of fathers with a youngest child aged 0–4 and 33% of fathers with a youngest child aged 5–11 (*ibid*). Distress rose for both unemployed and employed parents, from 20 to 36% in unemployed fathers, and from 7 to 27% in parents with a youngest child aged 5–11 (*ibid*). In terms of the numbers of people affected, there were estimated to be roughly 330,000 unemployed fathers, meaning about 120,000 unemployed fathers were experiencing “high mental distress”; and there were estimated to be nearly 1,500,000 parents of children aged 5–11, meaning close to 400,000 parents experiencing high mental distress (*ibid,* p. 4).

When it comes to explaining this increase, the report suggests it is not primarily a matter of financial stress when it comes to employed parents, because their reported rates of financial stress were similar to other parents’. More likely the explanation was “life changes beyond their family’s financial situation: namely, widespread working from home arrangements and school closures” (*ibid,* p. 5). An existing cohort of Australian mothers based in either Victoria or Tasmania with children aged between 5.9 and 7.2 years old was surveyed between May to December 2020. Of the 319 respondents, 85 reported that they had experienced job or income loss (27%), and 83 reported that a family member had experienced job or income loss (27%) (Bryson et al. 2021, p. 8). Half of the mothers (49%) chose “often” or “almost always” in response to an item asking “how difficult at home learning has made paid work/home duties for [the] mother” (*ibid*, p. 8 & Table 1).

What are the health impacts of stress? A literature review from 2017 notes that there has been research into the effects of stress on the nervous system since 1968 (so for over 50 years), with studies showing a range of impacts including “structural changes in different parts of the brain”, and “atrophy of the brain mass and decrease in its weight” (Yaribeygi et al. 2017, p. 1057). There are a range of impacts on memory (*ibid,* pp. 1059–1060), on cognition and learning (*ibid,* pp. 1060–1062), on immune system function (*ibid*, pp. 1062–1063), on cardiovascular system function (*ibid,* pp. 1063–1064), on the gastrointestinal system (*ibid,* pp. 1064–1066), and the endocrine system (*ibid,* p. 1066). Stress can cause changes in the brain that reveal themselves as “behavioural, cognitive, and mood disorders” (*ibid,* p. 1061). The American Psychological Association also include impacts on the male and female reproductive systems in the list of health impacts of stress.^27^

Increased incidence, frequency, or severity of stress may then be expected to correspond to increases in these negative health impacts, so to the degree that lockdowns are responsible for increasing stress, they should also be attributed the relevant proportional increase in the disease burden.

### Food and alcohol

In a global survey of 22,008 people aged 16–74 across 30 countries, administered between October and November 2020, Australia had the dubious honour of coming first in the world for increased alcohol consumption under COVID-19 restrictions (Bailey et al. 2021, p. 3). Reported in an article for *news.com.au*, “21% of Australians conceded they consumed more alcohol during the pandemic” (Priest 2021).^28^ 12% of Australians drank every day, more than half of the people whose drinking increased during lockdown continued drinking at higher levels after lockdown ended, and 37% said they intended to maintain this practice. The key drivers of this change were stress, home-schooling, anxiety, and unemployment (Priest 2021, reporting statistics from the Alcohol and Drug Foundation). A slightly greater number of women than men increased their alcohol consumption (18% compared to 16%), citing the management of stress as the main reason (Miller et al. 2021, p. 2, citing the Australian Institute for Health and Welfare).

A survey of 1,218 Australian women aged 45–64 in May 2020 found 316 women more likely to drink alone (30.6%), 246 women consuming more alcohol (23.8%), and 185 women stockpiling alcohol at home (17.9%) (Miller et al. 2021, p. 4, Table 1). The authors said that “Drinking more alcohol during COVID-19 was associated with nearly five times the likelihood of problematic drinking in Australian women”, and that “Stockpiling alcohol was associated with three times the likelihood of problematic consumption in Australian women” (Miller et al. 2021, p. 5). In the same study, the authors also note that “women are more susceptible than men to the many health impacts of alcohol and also more likely to develop alcohol disorders” (*ibid*, p. 2; citing Milic et al. 2018), and that “Alcohol has a dose-respondent relationship with the development of breast cancer, and has been identified as the biggest modifiable risk factor for breast cancer globally” (Miller et al. 2021, p. 2).

The long-term health impacts of alcohol depend on how much alcohol is consumed. In Australia, “the risk of dying from alcohol-related disease and injury remains below 1 in 100 if no more than 10 standard drinks are consumed each week and no more than 4 standard drinks^29^ are consumed on any one day”.^30^ Consumption of alcohol at higher rates can include impacts on mental health including increased risk of suicide; dependence on or addiction to alcohol; diabetes and weight gain; impotence and issues with sexual performance; several types of cancer; fertility issues (in men, reduced sperm count and reduced testosterone); brain damage; stroke and dementia; high blood pressure, heart damage, and heart attacks; and liver failure and cirrhosis of the liver.^31^

Increased alcohol consumption (particularly in men) also feeds into other impacts; the Australian Institute of Criminology identifies “increased alcohol consumption among domestic violence perpetrators” as a factor contributing to increases in both the prevalence, and the severity, of domestic violence perpetrated during the course of the pandemic (Boxall et al. 2020, p. 1).

35% of Australian respondents to the same global survey mentioned at the start of this section reported having gained weight (although 19% reported having lost weight) (Bailey et al. 2021, pp. 5 & 6). A national survey of 2000 + Australians aged 18–65 confirms this finding, with 41% saying they were snacking more throughout the day, 24% saying they had increased their ordering of takeaways from online delivery services, 37% saying they had gained weight 72% saying they were concerned about their weight, and 49% saying they were trying to lose weight.^32^

The International Agency for Research on Cancer, part of the World Health Organization, has linked excess body weight to 13 types of cancer.^33^ Being overweight can also lead to heart disease and stroke, type 2 diabetes, and musculoskeletal disorders.^34^ All of these “cause premature death and substantial disability” (WHO 2013).

### Quantity vs. quantity in sum

These three areas of mortality and morbidity impacts of policy responses to COVID-19 are not exhaustive. There are also mental health impacts on children, in having routines disrupted, being prevented from seeing extended family and friends, having reduced time outdoors, being prevented from using playgrounds (Tullock & Lawford-Smith 2021), and more generally, as Godfrey-Smith puts it, being raised “in an atmosphere of isolation and fear” (Godfrey-Smith 2021, p. 4).^35^ There are victims of trauma, abuse, and addiction whose recovery sessions have been ended or moved online, disrupting their progress and in some cases setting it back. *The New York Times* reported on a domestic violence survivor whose weekly therapy sessions had been moved online and whose support group was cancelled outright, who had subsequently turned back to “unhealthy coping mechanisms, like drinking and smoking” (Gupta and Stahl 2020). For the estimated 2.3 million Australians in single-person households (25% of the total households) (AIHW 2017), the consequences of extended lockdown might include social isolation, loneliness, anxiety and depression.^36^ There are health screening procedures that have been disrupted, and so diseases that may not be detected early when prevention and cure is most straightforward. For example, BreastScreen Australia conducted 30% fewer mammograms between January and June 2020 than in the same period prior to the pandemic (Miller et al. 2021). My aim here is not to cover all the possible impacts on quantity of life, but merely to note that there *are* extensive impacts.

Some proportion of these impacts are likely to have been caused by the pandemic itself, even if we had not locked down. As we saw in sub-Sect. 2.1, domestic violence has increased during economic downturns, terrorist attacks, and natural disasters. Larger numbers of deaths, caused by not locking down, would also have affected jobs and the economy. Worrying about getting sick is a source of stress, and people may have self-soothed with food and/or alcohol. I am assuming, I think reasonably, that these things were made considerably worse by the specific conditions of lockdown. For example, researchers comparing South Korea (no/minimal lockdowns)^37^ with the United States and United Kingdom estimated that “at most half of the job losses in the US and UK can be attributed to lockdowns” (Aum et al. 2021). Lockdown increases the exposure of victims of domestic violence to perpetrators, and the suspension of normal routines makes it harder for people to maintain healthy patterns of diet and exercise.

Once we take all these impacts seriously, it’s no longer obvious that lengthy lockdowns were the best available policy response to the COVID-19 pandemic. As of 4th November 2021, there had been 1768 total deaths from COVID-19 in Australia.^38^ An Australian Bureau of Statistics (ABS) analysis of excess deaths (for a definition see the start of Sect. 2) released in late November 2020 found only a small number of excess deaths from all causes (48 deaths), and a small number of excess deaths from specific causes, including pneumonia (22 deaths), diabetes (38 deaths), and dementia (10 deaths). The ABS note “international studies have cited potential misdiagnosis of COVID19, changes in access to healthcare and social isolation as possible reasons” for the cause-specific increases.^39^ That returns us to the point made earlier, that the excess deaths measure is useful in distinguishing a baseline of ‘normal’ deaths against which to compare deaths from COVID-19, but it comes with the problem that it will include deaths from the policy *responses* to COVID-19 too, including lockdowns. The ABS also noted “significantly lower than expected numbers of deaths … recorded from the end of May to mid-July 2020”, which was driven by lower numbers of deaths from “influenza, pneumonia and chronic respiratory conditions during that period”.

These are modest totals, but supporters of lockdowns will argue that they are so modest precisely *because of* lockdowns. To compare quantity (of death and disease from COVID-19) against quantity (of death and disease from extended lockdowns) we’d have to know the counterfactuals, namely what the total deaths, and excess deaths, from COVID-19 would have been without extended lockdowns.^40^ We should not assume that life without lockdowns means ‘business as usual’: it is not unlikely that it would involve a substantial amount of voluntary behavioural change in response to governmental advice (see also discussion in Allen 2022, esp. pp 13–15). I will not hazard to turn the impacts outlined in Sects. 2.1–2.3 into an approximate number of disability-adjusted life years, nor attempt to defend a specific counterfactual that could be used as the comparison quantity (although see fn. 41 for ballpark calculations). My point here is simply that there were significant and substantial quantity impacts of lockdown. Together with the significant and substantial quality impacts, we may get closer to the conclusion that we lost as much, or more, as a society by implementing extended lockdowns than we would have by not locking down—or, as Godfrey-Smith suggests, by only locking down for “short resets” (Godfrey-Smith 2021, p. 4).

## Quality vs. quantity within locked-down populations

Australian journalist Gideon Haigh wrote recently in *The Australian*:

For the past 18 months, I have been taking the same walk through the same Melbourne suburban streets in the same direction at roughly the same time every day. I’ve thought at times of varying it but always refrained. It wasn’t a pleasure, nor was it a “freedom”, except in this word’s modern sense as a privilege granted by a premier. So I wasn’t prepared to perform it other than mechanically, in precisely the mean and grudging spirit of its permission. I’m well aware this sounds perverse. It is perverse. I don’t care. We each had a way of coping with the world’s most protracted lockdown, and this was mine, with an interior monologue of quiet seething to match (Haigh 2021).

Melbourne’s lockdown was severe. The University of Oxford’s Blavatnik School of Government developed a Stringency Index which ranked countries on the restrictiveness of their lockdowns, and ranked Australia at 78 (where 100 is the most extreme). They ranked countries only, but *The Age* reported that “rough calculations using the methodology showed the city’s [Melbourne’s] stringency rating would have topped out in the mid 1990s” (Hope 2021). At its most restrictive point, Melbourne residents had to be inside their homes between 9 pm and 5am; could not travel outside of a 5 km zone from their houses; could not leave their houses for more than 1 h a day; could leave their houses only for a limited number of reasons, including exercise, caregiving, authorised work, and shopping for groceries and essentials (only one person per household was allowed to do this). Gyms were closed, outdoor gatherings were severely limited, playgrounds were closed, funerals were limited. Travel between states was mostly not permitted, even for funerals.^41^ Haigh’s response, as he notes, may seem perverse; minimizing rather than maximizing pleasure within the available constraints. But it is also understandable, a form of self-expression, a way of exercising control while being tightly controlled by the state government.^42^

Melbourne’s total number of days in lockdown (through six separate lockdowns) was 262,^43^ almost nine months, making it “the world’s most locked-down city” (Al Jazeera 2021). Melbourne’s first lockdown started on the 30th of March 2020, and between the six separate lockdowns there were gaps of 57 days, 107 days, 99 days, 35 days, and 86 days, respectively (Dunstan 2021; BBC News 2021). Many workplaces kept people working from home in these gaps, many universities kept students away from campus. So some people have been working and studying from home for 19 months (April 2020 through October 2021), some cohabiting with others in the same position (partners, housemates), some attempting to facilitate their kids’ online learning at the same time. Parents could only take their kids out for an hour a day, and not to the playground, because they were closed.

Haigh commented on his quality of life under Melbourne lockdown explicitly: “It was, just, bearable. You could get by, providing you expected nothing good to happen, everything to take twice as long as it should, and no useful end to be served. Like a body adapting to starvation, you rationed expectation, postponed pleasure, concentrated on the little you could control in your unkempt lethargy, and thought sympathetically of the worst-off, if in an abstract sense” (Haigh 2021).

Much about severe and extended^44^ lockdowns decreases quality of life. Godfrey-Smith commented on a number of such things: “the effects of economic dislocation and unemployment, also the effects of disrupted education,^45^ with each of these having stark consequences for inequality” (Godfrey-Smith 2021 p. 5); the “suppression of liberty and autonomy” (*ibid,* p. 2); the shrinking of longer-term opportunities and the reduction of human contact, especially near the end of life (*ibid*); bankruptcies (*ibid,* p. 4); “the consequences of raising children in an atmosphere of isolation and fear” (*ibid*); increases to social inequality, including racial inequality, caused by disruption to education (eventually impacting the ‘meritocracy’) (*ibid,* p. 6); ‘the socialization of young people in the years before school, unemployment and small business failure, mental health’ (*ibid*); a widened gap between the rich and the poor (*ibid,* p. 9); and changed relations between people and police forces because of the over-policing trivial things (*ibid,* p. 13).^46^

Godfrey-Smith focuses on reductions in quality at three particular stages in life. Children experience isolation and fear, have their educations disrupted, and miss out on important socialization. Young people, “people who have left school, and are in their late teens and 20 s” (*ibid,* p. 16), are substantially delayed or disrupted at a crucial period in which they would normally be choosing careers and beginning meaningful relationships. They experience loss of direction and mental health impacts. And old people are impacted in particular by reduced human contact. What he says about reduced contact near the end of life is particularly stark:

In the view of many, it is a disaster to die alone, and also to face alone, over many months, what feels likely to be the last part of one’s life, or a large part of the last stages. For many old and infirm people, companionship and contact with loved ones are fundamental, and much of what it’s worth being alive *for*. Some extra risks are worth taking if it means you can stay in contact with people who make your life meaningful. But this choice has been taken out of many people’s hands. The decision not to allow visitors in aged care homes and many hospital settings has surely been the source of a great amount of unseen despair and misery over the course of the pandemic (*ibid,* p. 15).

Apart from these more serious negatives, lockdowns also simply caused a huge loss in specific pleasures—the pleasures of meeting friends, spending time with extended family, going out: to restaurants, cafes, bars, the cinema, the theatre, comedy shows, concerts, sports games, and other kinds of events; going out in nature, on hikes, to mountains, to the beach. Many of the things we think contribute to meaningful lives were prevented, disrupted, put on hold: making new friends (e.g. when starting university, or a new job), dating, strengthening or solidifying relationships with extended family, with existing friends, with colleagues (difficult if not impossible to accomplish by Zoom or Teams). Even for someone without the negatives of financial stress, or family or relationship stress, spending 19 months at home, only going out for an hour or so of exercise or grocery shopping, is a life without many positives. The uncertainty of when lockdown would end, and the continually frustrated expectations of an end (with ever-extending deadlines), also disrupted *hope*.

Is it fair to say that lockdown life, at least for many people, was Parfit’s ‘drab life’? We had more than muzak and potatoes, even at the worst of times. As to food and drink, for those who could afford it much of what was available pre-lockdown was still available for delivery, simply to be consumed inside the same walls we were all trapped inside of, and without the company of anyone not living within those walls. All the music, comedy, sport, movies, and television shows were still there, just mediated by a screen, rather than to be experienced live, as part of an audience or crowd. Freedom was not *entirely* curtailed; if there was nature inside your 5 K zone, then you could have nature, for an hour a day (so long as you experienced it in a way that passed as exercise: no sitting about reading a book). People could, obviously, still find meaning: still read great novels, still master new skills (taking up new hobbies or picking up old ones, at least those whose resources could be ordered online and delivered, and which could be conducted within one’s home—so yes to learning how to build shelves, and no to taking up rock-climbing).

There is a lot of constitutive luck in how drab lockdown life was. The single-household extroverts lost a lot more than the single-household introverts, but the multi-person household introverts lost a lot more than the multi-person household extroverts.^47^ The sporty and adventurous lost a lot more than the bookworms. The overworked-but-desperately-wishing-for-a-hiatus-to-write-that-novel did better than the overworked-with-no-idea-what-to-do-with-more-time.

Perhaps rather than asking ‘was lockdown life worth living?’ and answering, like Haigh, ‘just barely’, the better question is, did each of us, as individuals, have reason to choose quantity (of our own lives—reducing the risk to our own mortality and morbidity) over quality of life? If we didn’t—or if most of us didn’t, and wouldn’t have chosen that if given the choice—then that would appear to create a challenge to the fact that lengthy lockdowns were imposed on us. That would be the state paternalistically intervening to impose the value of preservation of quantity of life (and from a specific cause, namely COVID-19, only) having strong priority over *both* preservation of quantity of life from other causes (here specifically lockdowns), and preservation, or minimization of decrease, in quality of life. To the extent that this policy-making decision was underpinned by an assumption that this is what a fully-informed population would choose, it would then be a bad policy.

Even if we didn’t yet have reason to prefer quality over quantity (for our own lives), it’s worth asking ourselves how long lockdown could go on before we considered ourselves to be living a version of the Repugnant Conclusion. When I’ve spoken to friends about this, answers have varied widely: 5 years is a clear case; maybe 2.5 years, but less if you’re younger; 2 years, because we can expect to have developed and administered a vaccine by then; 1 year—we’ve been in the Repugnant Conclusion for a while already. Parfit taught us that we don’t always have a reason to prefer population increase over quality of life for each individual, even where that would increase the total population wellbeing. We need to think more carefully about the inverse: whether we always have a reason to prefer population *maintenance* (non-decrease) over quality of life for each individual, even where that would *decrease* the total population wellbeing.

## Footnotes

1 There is room to argue here about whether what had strong priority in countries with strict and lengthy lockdowns was the preservation of existing lives given the threat from COVID-19, or was rather the protection of the healthcare system (particularly hospitals) from being overwhelmed. The latter has a justification in terms of the preservation of existing lives, too, but the former is more directly tied to COVID-19 deaths while the latter is about death and disease from all causes.

2 I’m following the understanding of lockdowns used by the Blavatnik School of Government at the University of Oxford in their COVID-19 Government Response Tracker. They create a ‘stringency index’ by country, based on nine indicators including school closures, workplace closures, the cancellation of public events, restrictions on gatherings, the closing of public transport, public information campaigns, stay at home requirements, restrictions on domestic travel, and restrictions on international travel. https://www.bsg.ox.ac.uk/research/research-projects/covid-19-government-response-tracker (see the ‘sources’ tab in the ‘visualisations’ section).

3 Or as authors in the *British Medical Journal* put it: “Despite increasing evidence on the unintended, adverse effects of public health interventions such as social distancing and lockdown measures, there are few signs that policy decisions are being informed by a serious assessment and weighing of their harms on health” (Bavli et al. 2020).

4 Page numbers correspond to the Oxford Scholarship Online version of the book.

5 Or in an earlier paper, “There might be worse housing, overcrowded schools, more pollution, less unspoilt countryside, fewer opportunities, and a smaller share per person of various other kinds of resources” (Parfit [1986] 2004, p. 8).

6 In one historical example of the former, in 1972 the Ugandan President Idi Amin ordered the expulsion of most of the roughly 80,000 Indians, Pakistanis, and Bangladeshis living in Uganda, claiming that they were ‘bloodsuckers’ on the economy. http://news.bbc.co.uk/onthisday/hi/dates/stories/august/7/newsid_2492000/2492333.stm. Indians owned ‘nearly all the large shops and businesses’, which created resentment in African Ugandans, which Amin was able to exploit to his own aggrandizement. At a meeting attended by the international media, he ‘launches into an attack on the Asian community, accuses them of tax evasion, funnelling money out of the country, and discriminating against black Ugandans in business. In other words, economic and cultural treason’ (*How to Become a Tyrant* (2021)*,* Season 1, Episode 3). Black Ugandans who supported the expulsion were likely to have believed that it would increase their own quality of life.

7 Cf. fn. 2 – I won’t mention this again, but it should be taken to remain an alternative whenever I talk about this as the strong priority for countries with strict and extended lockdowns.

8 I mean this in the colloquial sense of residing in Australia during the pandemic, not the technical sense of having a visa status as temporary or permanent resident.

9 https://data.worldbank.org/indicator/SP.DYN.LE00.IN?locations=AU.

10 The sex-distinguished expectancies are for children born between 2017 and 2019. https://www.aihw.gov.au/reports/life-expectancy-death/deaths-in-australia/contents/life-expectancy.

11 Strictly speaking, he talks in terms of ‘lexical superiority’, see e.g. p. 112.

12 In an earlier paper, he had written “The people in Z never suffer; but all they have is muzak and potatoes” (Parfit [1986] 2004, p. 9). In the later paper, Parfit acknowledges that different people will have different qualities of life were the “muzak and potatoes” circumstances to become actual. He writes “Even if we lost most external goods, some of us would have inner mental resources with which we could make our lives fairly good, by composing long poems, for example, or thinking about some intellectual problems. Some political prisoners have lived fairly good lives while they were in solitary confinement. But some other people would find lives in such conditions worse than nothing” (Parfit 2016, p. 118).

13 To be clear, I think this should provide motivation to governments, non-governmental organizations, and individual philanthropists to work to increase such people’s quality of life; aside from being intrinsically bad for those who must live such lives, it is a terrible waste of human potential.

14 Page numbers are to the online-first PDF of Godfrey-Smith’s paper in this volume, online at https://link.springer.com/article/10.1007/s40592-021-00140-6.

15 For tractability, I am treating death and disease together as a reduction in quantity of life, given that disease may shorten life and create a risk to life, even though some non-terminal diseases will do neither. This is a familiar approach, taken in talk of DALYs, or ‘disability-adjusted life years’. (The alternative would have been to treat *some* disease as a reduction in quality of life only, but that would have required separating out different types of disease). Everything I discuss in this section as negatively impacting quantity of life should also be understood to reduce quality of life, but I won’t double-up in discussing them, focusing in Sect. 3 more on the experiences that contribute to how meaningful and/or enjoyable life is.

16 In her book *See What You Made Me Do* (2019), investigative journalist Jess Hill follows Yasmin Khan in using the term ‘domestic abuse’ rather than ‘domestic violence’, on the grounds that ‘in some of the worst abusive relationships, physical violence is rare, minor or barely present’ (Hill 2019, p. *vii*). Others prefer ‘intimate terrorism’ (Taub 2020). I will use ‘domestic abuse’ except where I’m quoting others.

17 France’s spike was reported in the same week the *Times* article came out (6th April 2020), but the article does not give the period in which the spike occurred, or the period taken as the baseline.

18 ‘Coercive control’ was understood to mean ‘they experienced three or more forms of emotionally abusive, harassing and controlling behaviours in the three months prior to the study’ (Boxall et al. 2020, p. 7).

19 The wording of this in the Bulletin is slightly confusing: ‘when the sample was limited to women who had been in cohabiting relationships in the previous 12 months’. On its face, this would seem to distinguish women who had cohabited with a partner at *any* point in the past 12 months from women who had not. But domestic violence is understood in the survey as violence perpetrated by a current or previous cohabiting partner. So it could not happen that “4.2 percent of all women and 8.2 percent of women in cohabiting relationships experienced physical violence” (Boxall et al. 2020, p. 6). Thus I interpret it to mean, women who had been living with their partners for the last full year.

20 https://esperanzaunited.org/en/is-this-abuse/.

21 The ‘burden of disease’ is the gap between the actual health of a population and the ideal health of that population. Ideal health is understood as ‘every individual living without disease or injury to the theoretical maximum life span’. The analysis of disease burden combines both the number of years lost to premature death, and the number of *healthy* years lost to living with poor health. See discussion at https://www.aihw.gov.au/reports/australias-health/burden-of-disease.

22 https://www.aihw.gov.au/reports/australias-health/health-impacts-family-domestic-and-sexual-violence.

23 I’m focusing here on increases to work and financial stress, but it’s worth noting that there are likely to be at least some Australians for whom lockdown conditions reduced work and financial stress, for example as a result of reduced commute time, reduced expenses, and reduced interpersonal conflict (including reduced exposure to bullying and sexual harassment in industries with high rates of each, such as the legal industry—see Pender 2019). Given the number of job losses, reductions to income, and families trying to balance working from home with parental responsibilities, it is unlikely that these benefits counterbalance the harms.

24 https://www.abs.gov.au/articles/one-year-covid-19-aussie-jobs-business-and-economy.

25 Two Victorian women I spoke to facing loss of income as a consequence of refusing mandatory vaccination said that the support payments wouldn’t have covered their rent alone (Lawford-Smith 2021).

26 They understood ‘mental distress’ as feeling nervous and/or depressed ‘all of the time’, or ‘most of the time’ (in the four weeks prior to a survey being administered); and ‘financial stress’ as people reporting ‘very financially stressed’ or ‘moderately financially stressed’ when asked about their current circumstances in paying for essential goods and services (e.g. rent, mortgages, bills) (Melbourne Institute 2021, p. 8).

27 https://www.apa.org/topics/stress/body.

28 The global survey also reports 8% of Australians as decreasing their alcohol consumption.

29 A bottle of beer and a glass of wine are each between 1 and 1.6 standard drinks depending on the size/serve. https://www.health.gov.au/health-topics/alcohol/about-alcohol/standard-drinks-guide.

30 https://www.health.gov.au/health-topics/alcohol/about-alcohol/what-are-the-effects-of-alcohol.

31 *ibid.*

32 Survey participants were asked about the period February to July 2020. https://www.cancerwa.asn.au/articles/news-2020/unintended-covid-consequences-over-a-third-of-auss/.

33 https://www.cancerwa.asn.au/prevention/weight/body-weight-and-cancer-risk/.

34 https://www.who.int/news-room/q-a-detail/obesity-health-consequences-of-being-overweight.

35 Alexis Madrigal describes his 8 year-old child’s reaction when he (Madrigal) got COVID-19 as ‘so mad and maybe so scared that they could barely look at me’ (Madrigal 2021).

36 In Victoria for the first two lockdowns, people in sexual relationships were allowed to form a bubble and visit each other’s households, but people in other kinds of dyadic relationships—friendships, family, colleagues, acquaintances—were not (see discussion in Collins & Ferracioli 2020).

37 See also (Godfrey-Smith 2021, p. 9 fn. 8).

38 https://www.health.gov.au/news/health-alerts/novel-coronavirus-2019-ncov-health-alert/coronavirus-covid-19-case-numbers-and-statistics.

39 https://www.abs.gov.au/articles/measuring-excess-mortality-australia-during-covid-19-pandemic#discussion.

40 The UK and Australia in May 2020 had very different case numbers, but equally stringent restrictions according to the Oxford Stringency Index (Miller et al. 2021, p. 3). But the two countries also have different population sizes and income levels. The AIHW compared Australia’s case and death rates to ‘comparable countries’, understood as countries with a similar proportion of people over the age of 65, similar population health (indicated by life expectancy), and similar health care systems and public spending on health care (AIHW 2021, p. 61). The countries were Canada, Sweden, United Kingdom, and New Zealand. This was useful because these countries had a range of different approaches to lockdowns. If Australia had had the same rate of cases as four of those countries (all except New Zealand) (all adjusted for population size), then we would have had between 15 × and 46 × as many deaths as we did have (if we had the same rate of cases as New Zealand, adjusted for population size, then we would have had substantially fewer deaths) (*ibid,* pp. 61–62). The relationship between lockdown stringency and fewer deaths is not linear. If Australia had had Sweden’s rate of cases, it would have had more cases than if it had the United Kingdom’s rate, but it would have had more deaths with the UK’s rate than with Sweden’s (*ibid*). 15 × as many deaths would be 26,520; 46 × as many deaths would be 81,328. If the distribution across the age cohorts remained the same, then 59% of these people would be past the age of life expectancy, and 41% below it. That gives us a very rough range of deaths before the age of life expectancy, without lockdowns, as being between 10,873 and 33,344.

41 A student in my department was denied permission to travel from Victoria to Tasmania for his father’s funeral (Prien 2021). There’s also an impact of closed international borders. Some foreigners trapped inside Australia faced a lack of government support, insecure residency status, racism, and severely interrupted life plans.

42 Jean Améry writes ‘I finally relearned what I and my kind often had forgotten and what was more crucial than the moral power to resist: to hit back. Before me I see the prisoners foreman Juszek, a Polish professional criminal of horrifying vigor. In Auschwitz once he hit me in the face because of a trifle; that is how he was used to dealing with all the Jews under his command. At this moment—I felt it with piercing clarity—it was up to me to go a step further in my prolonged appeals case against society. In open revolt I struck Juszek in the face in turn. My human dignity lay in this punch to his jaw—and that it was in the end I, the physically much weaker man, who succumbed and was woefully thrashed, meant nothing to me. Painfully beaten, I was satisfied with myself. […] In the punch, I was myself—for myself and for my opponent’ (Améry 1980, pp. 21–22). Punching the guard did not improve Améry’s circumstances, but it was nonetheless something it was important for him to do, because it was a way to exercise control, to choose, to have dignity.

43 North Melbourne had an additional 7 days, for a total of 269 days.

44 As Godfrey-Smith says, “As with the disruption of schooling, the duration of the suspension matters. Measures that have limited importance when they apply over a two-week period are a different matter when extended for months, and when they are reintroduced repeatedly” (Godfrey-Smith 2021 pp. 13–14).

45 One specific version of disrupted education is that affecting international students who are second-language speakers. A student I spoke with said she had been relying on the full immersion experience to improve her English language skills (above that required for entry), and that Zooming into classes occasionally from her home country was not remotely sufficient to this. This will have knock-on effects: the quality of her assignments is likely to be lower, and lower grades will affect her career opportunities after university; she will also have reduced opportunities more generally relative to what fluency in English may have offered (separate from her degree). In 2019, pre-pandemic, Australia had 440,667 international students (Ferguson & Spinks 2021). Of course some of these will already have English as a native language, or will already be fluent in English. But those who do not, and were prevented from entering the country, are likely to experience negative impacts from online learning.

46 In her October 2021 interview prior to quitting the Victorian Police, Krystle Mitchell commented on perceiving a change in the public attitude toward the police as a result of their policing of the pandemic restrictions. https://www.youtube.com/watch?v=0Kn6AFl5G1c.

47 These are impacts we’re not used to taking very seriously, but both may have impacts on mental health—feeling suffocated/claustrophobic when you need space (for introverts pushed together in close proximity with partners, family members, or housemates), or feeling lonely/isolated when you need company (for extroverts living alone, or who may have partners, family members, and friends living in other states they can’t travel to see).
